# The Potential of a Protein Model Synthesized Absent of Methionine

**DOI:** 10.3390/molecules27123679

**Published:** 2022-06-08

**Authors:** Ronald J. Savino, Bartosz Kempisty, Paul Mozdziak

**Affiliations:** 1 Prestige Department of Poultry Science, College of Agriculture and Life Sciences, North Carolina State University, Raleigh, NC 27695, USA; bkempisty@ump.edu.pl (B.K.); pemozdzi@ncsu.edu (P.M.); 2 Department of Anatomy, Poznan University of Medical Sciences, 60-781 Poznan, Poland; 3 Department of Histology, Poznan University of Medical Sciences, 60-781 Poznan, Poland; 4 Department of Veterinary Surgery, Institute of Veterinary Sciences, Nicolaus Copernicus University, 87-100 Toruń, Poland

**Keywords:** methionine, cysteine, antioxidant, protein, cell, stability, protein synthesis

## Abstract

Methionine is an amino acid long thought to be essential, but only in the case of protein synthesis initiation. In more recent years, methionine has been found to play an important role in antioxidant defense, stability, and modulation of cell and protein activity. Though these findings have expanded the previously held sentiment of methionine having a singular purpose within cells and proteins, the essential nature of methionine can still be challenged. Many of the features that give methionine its newfound functions are shared by the other sulfur-containing amino acid: cysteine. While the antioxidant, stabilizing, and cell/protein modulatory functions of cysteine have already been well established, recent findings have shown a similar hydrophobicity to methionine which suggests cysteine may be able to replace methionine in all functions outside of protein synthesis initiation with little effect on cell and protein function. Furthermore, a number of novel mechanisms for alternative initiation of protein synthesis have been identified that suggest a potential to bypass the traditional methionine-dependent initiation during times of stress. In this review, these findings are discussed with a number of examples that demonstrate a potential model for synthesizing a protein in the absence of methionine.

## 1. Introduction

The understanding of the scope of methionine’s function has been a subject of much debate for a number of decades. It is often considered an essential amino acid due to its inability to be synthesized in vivo as well as its necessity for the initiation of protein synthesis. Beyond that, its stabilizing role in the hydrophobic core of proteins was believed to be replaceable by any other hydrophobic amino acid. Unlike methionine, cysteine has been explored as a multifunctional amino acid that has a number of properties regularly used by cells. Besides cysteine being the rate-limiting component of the well-known antioxidant glutathione [1], the antioxidant function of cysteine also became commonly known as an endogenous protection mechanism used by proteins. Cysteine was also identified for its stabilizing ability in endogenous and exogenous proteins as well as its participation in certain signaling pathways of cells [2,3,4,5,6,7,8,9,10].

Within the past two decades, groundbreaking research has emerged that has revealed a number of newly discovered properties of methionine that expand its role in proteins and within cells well beyond the previous assumptions. An in-depth review by Rodney Levine and co-workers characterized many of the unique and novel aspects of methionine, giving way to further exploration into its importance outside of protein synthesis [11]. Unique interactions formed by methionine granted to the amino acid by its inclusion of a sulfur atom have been characterized and found to contribute to the stability of intracellular proteins [12,13]. Additional work has shown similar antioxidant properties to that of cysteine that serve important protection functions within proteins [14], with the reduction of methionine antioxidant activity being related to cell aging and certain diseases [15,16]. Methionine has also shown an ability to serve as a modulator of cellular activity during times of oxidative stress to prevent further cell damage [17]. With a clearer picture of the array of roles that methionine has for protein and cell function, the focus shifts into understanding whether these functions are essential. New research looking into the properties of cysteine reveals a similar hydrophobicity to methionine [18] as well as an ability to form protein stabilizing S-aromatic motifs, which has been previously identified. As it seems chemically justifiable that cysteine may be able to take the place of methionine in the role of protein stabilization, redox signaling, and antioxidant function, the essential function of methionine may lie within the initiation of protein synthesis. Recently, novel research in the field of eukaryotic and bacterial protein translation has provided evidence for the initiation of protein synthesis with alternative amino acids and initiator tRNAs as well as the complete bypassing of the initiation stage by use of unique mRNA structures. In this review, the aforementioned functions of methionine are inspected in the context of their discovery and commonality. The potential proteins to remain functional after substitution of methionine with cysteine and for those proteins to continue supporting normal cellular activity is discussed based on the shared attributes between methionine and cysteine as well as recent evidence contesting methionine’s essential nature in initiating protein synthesis. 

## 2. Methionine’s Role in Protein Structure

**Figure 1:**
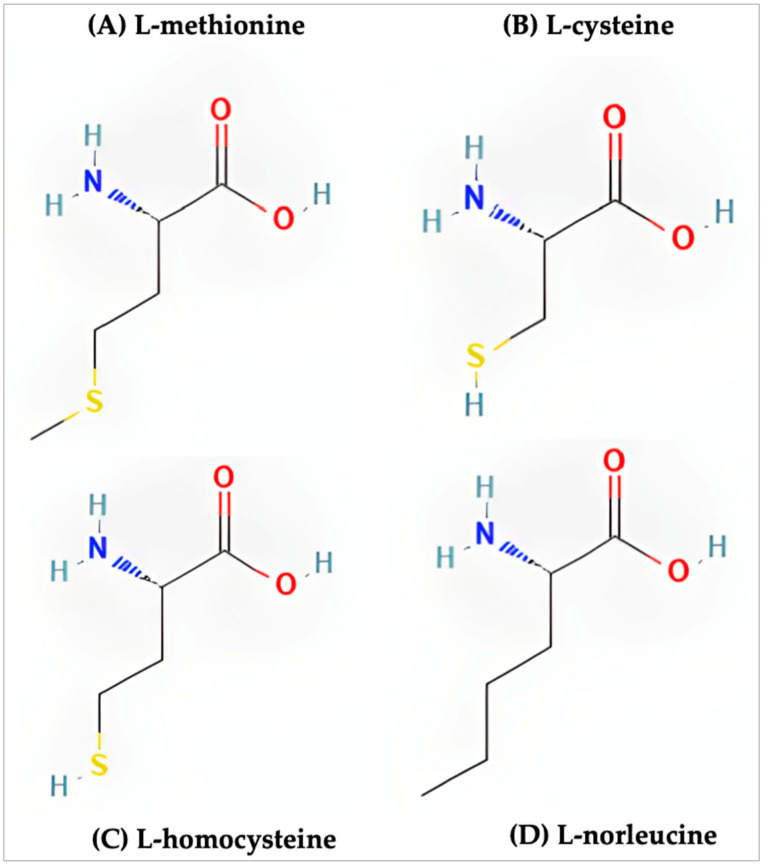
Structure of the sulfur-containing amino acids. (**A**) l-methionine (PubChem CID 6137), (**B**) l-cysteine (PubChem CID 5862), (**C**) l-homocysteine (PubChem CID 91552), (**D**) l-norleucine (PubChem CID 21236).

Methionine resides among a unique class of amino acids due to its incorporation of a sulfur atom. Along with methionine, cysteine and homocysteine share similar chemical structures (Figure 1). Of these three sulfur-containing amino acids, only methionine and cysteine play a role in the synthesis and structure of proteins. Early studies have shown that supplementing the diets of animals with only methionine recorded rates of growth similar to animals fed diets supplemented with both cysteine and methionine while animals fed diets supplemented with exclusively cysteine saw significantly lower rates of growth [19]. In follow-up studies it was shown that, at suboptimal levels of methionine, cysteine was able to compensate for this lack of methionine and normal rates of growth were observed [20]. These early findings, in the context of what is now understood of methionine, suggest that the essential nature of methionine is within its role during protein synthesis as the initiating amino acid.

What is commonly acknowledged is that methionine is both the first amino acid added during protein synthesis as well as regularly added to the growing peptide chain. To introduce the necessity of methionine in protein structure, the initiating methionine, and internal methionine will be described separately.

**Figure 2:**
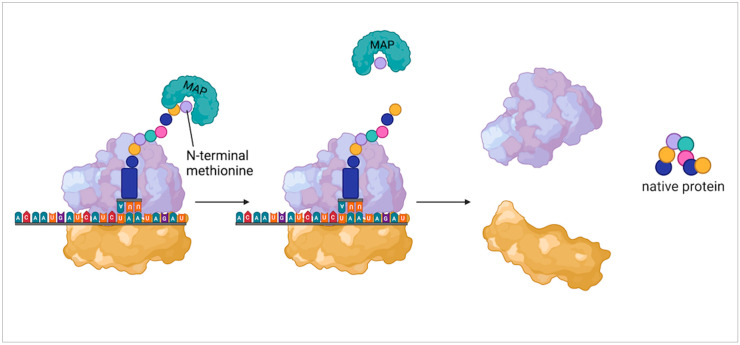
Methionine aminopeptidase acting on N-terminal methionine. This shows general function of methionine aminopeptidase (MAP) in eukaryotic protein synthesis. From left to right shows the binding of MAP to the N-terminal methionine, the removal of the methionine, and the disassociation of the ribosomal subunits leaving the native protein.

In eukaryotes and archaea, the initiating tRNA encodes for L-methionine while bacteria initiate translation with *N*-formylmethionine tRNAs. Though crucial for the initiation of protein synthesis, shortly after the onset of elongation this same methionine is removed from the polypeptide chain. As discussed by Wingfield [21], N-terminal methionine in eukaryotes is co-translationally cleaved by the enzyme methionine aminopeptidase (MAP) soon after it initiates protein synthesis (Figure 2). A similar mechanism can be seen in bacteria which first requires the removal of the formyl group by formylmethionine deformylase which leaves an N-terminal methionine ready to be removed via MAP. In any given proteome, about two-thirds of the proteins represent potential targets for MAP function and in bacteria and yeast, the inhibition of MAP activity is lethal. Other experiments have further shown that the removal of the N-terminal methionine or N-formyl methionine is essential for the function and stability of proteins [22,23,24]. So not only is N-terminal methionine not essential for the structural integrity of proteins, but its removal is also a necessity in many cases.

**Figure 3:**
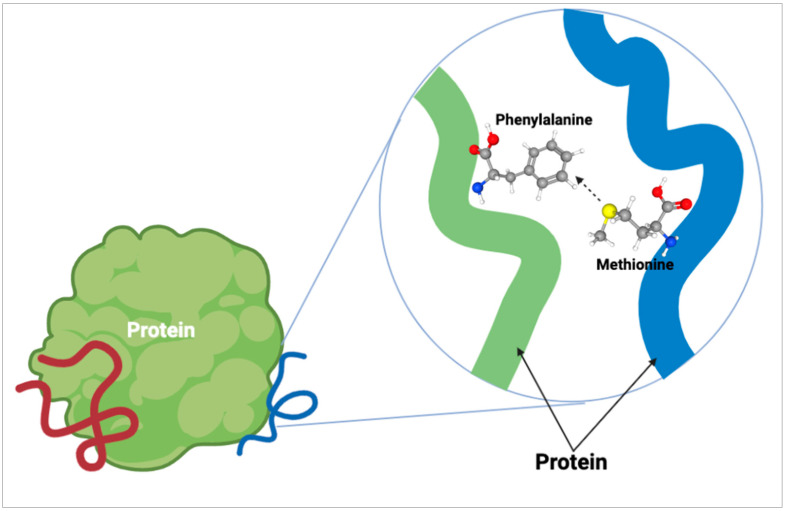
Methionine S-aromatic motif. The formation of an S-aromatic motif within proteins occurs between the sulfur atom (yellow ball) of amino acids such as methionine and an aromatic residue, such as phenylalanine, within proteins. These interactions are quite common within proteins and aid in stabilization.

As for methionine within the protein structure, our understanding is continually evolving as new findings emerge. Since methionine is a hydrophobic amino acid, it has often been contested that its main role in proteins is maintaining the structure of the hydrophobic core. It was believed for many years that other hydrophobic amino acids could replace methionine within a protein with little consequence. More recently, the role of the sulfur atom of methionine has been explored for its ability to form stabilizing bonds within a protein. Motifs known as S-aromatic motifs (Figure 3) are formed by the sulfur atom found in the side chain of methionine and aromatic residues within the protein which have been characterized as far back as 1985 [25]. Almost 20 years later, the protein-stabilizing contribution of S-aromatic motifs was shown by Randy Zauhar and colleagues [12] leading to additional research into the motifs [13]. The findings characterized these methionine-aromatic interactions as occurring at a greater distance than salt bridges (5–7 Å and 4 Å respectively) while the energies of these interactions are comparable. Work by Orabi and English [26] modeled S-aromatic motifs in proteins which showed that the redox capability of these interactions provides dynamic flexibility to proteins which is an important feature for their activity. As S-aromatic motifs are quite common within proteins, it is likely that they represent a significant stabilizing feature of proteins.

The oxidation of methionine to methionine sulfoxide is discussed later is another important function of methionine; however, how this oxidation affects S-aromatic motifs within proteins is very interesting. It was later discovered [27] that oxidation of methionine actually strengthens the methionine-aromatic interaction rather than weakening it. It is also worth noting that with increased rates of methionine oxidation, changes in the protein structure including increased protein surface hydrophobicity occur which can be an age-related effect occurring over time or during specific periods of oxidative stress [28]. Clearly, methionine’s capacity within proteins extends beyond merely contributing to the hydrophobic core of proteins; its stabilizing function should not be discounted.

## 3. Antioxidant Function of Methionine

### 3.1. Discovering Methionine’s Antioxidant Function

**Figure 4:**
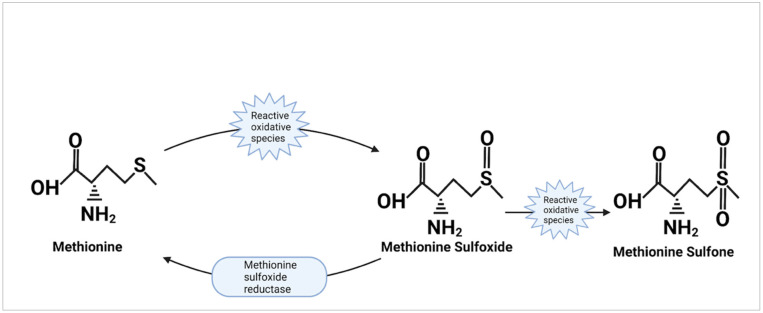
The oxidation and reduction of methionine. This displays the action of methionine as an antioxidant. Reactive oxidative species oxidize the sulfur side chain of methionine to produce methionine sulfoxide and may further oxidize methionine sulfoxide to produce methionine sulfone. The action of methionine sulfoxide reductase to reduce methionine sulfoxide back into methionine is also shown; though it is unable to reduce methionine sulfone.

Within certain proteins, methionine residues may be exposed to the outer surface, making them susceptible to interaction. Research completed nearly 40 years ago described the oxidation of methionine which produces methionine sulfoxide and, when further oxidized, produces methionine sulfone [29]. It was also observed that free and protein-bound methionine sulfoxide could be reduced back to methionine by methionine sulfoxide reductase while methionine sulfone was unable to be reduced (Figure 4). The oxidative capacity of methionine gave birth to the hypothesis that cells could utilize this property as an endogenous antioxidant for protection. Testing of this hypothesis began by observing the effect of exposing glutamine synthetase from *E. coli* to hydrogen peroxide [14]. The results showed that methionine residues on the surface of the protein that were susceptible to oxidization were arranged in a way that prevented the oxidation of the active site on the protein. Additionally, it was postulated that due to the ability to reduce oxidized methionine residues using methionine sulfoxide reductase, these methionine residues could protect against oxidization repeatedly [30]. Confirmation of the methionine antioxidant hypothesis was later done by Shen Luo and Rodney Levine in an experiment where methionine was substituted with norleucine in *E. coli* [31]. While enzymatic activity or growth rates were not significantly different in colonies supplemented with norleucine vs methionine, when exposed to oxidizing agents, cells with norleucine substitution died at rates significantly faster than those containing methionine. Later testing of the oxidative function of methionine found that cells increased methionine incorporation within proteins in times of oxidative stress [32]. A review by Rodney Levine and collaborators [11] looked even further to the adapted antioxidant function in the context of α-2-macroglobulin; a protein that was found with methionine residues lining its active site as a purposeful protection mechanism. What this implies is that proteins may have evolved to incorporate methionine residue in particularly vulnerable positions highlighting the importance of methionine as an endogenous antioxidant.

### 3.2. Reversible Oxidative Ability of Methionine

As important to the endogenous antioxidant ability as methionine are the enzymes that give methionine its repeated oxidative capacity. The oxidation of methionine to methionine sulfoxide yields both an R-MetO and S-MetO form which requires a different set of enzymes for their reduction [33]. The enzyme methionine sulfoxide reductase A (MsrA) is responsible for reducing S-MetO [34,35,36] while MsrB reduces the R-MetO form [37]. Though MsrA is able to reduce both free and protein-bound methionine, there are two separate enzymes that are needed to reduce R-MetO. MsrB reduces the protein-bound R-MetO but has low activity on free R-MetO; it is the enzyme-free methionine-R-sulfoxide reductase (fRMsr) that has high specificity and activity on free R-MetO [38,39,40]. Interestingly, the activity of fRMsr has only been observed in certain prokaryotic organisms. The reduction of methionine sulfoxide by MsrB is a process also dependent on yet another important protein: thioredoxin. During the reduction of R-MetO, an intramolecular selenide-sulfide bond is formed on the MsrB which is the enzyme in its oxidized state. To return the reducing capacity to MsrB, thioredoxin forms an intermolecular complex with oxidized MsrB which is subsequently resolved by thioredoxin resolving cysteine 35. At this point MsrB is back in its reduced state while thioredoxin is now oxidized, but the oxidized thioredoxin is quickly reduced by thioredoxin reductase thus completing the redox cycle of MsrB [41].

The importance of these enzymes has been noted in experiments where knocking out MsrA in mice [42] and bacteria [43,44] led to states of oxidative stress. In cases where the expression of MsrA was over-expressed, human T-cells showed increased resistance to oxidative stress [45] and was even found to double the lifespan in Drosophila [46]. The activity of these enzymes since their discovery has also been known to decrease with age and in certain diseased states [15,16,47,48]. Many new therapies have emerged that have focused on the antioxidant function of methionine and the reducing enzymes to counter increased oxidative states present during aging and disease [49,50,51,52]. These findings reveal that the role of methionine oxidation and subsequent reduction by these enzymes is not only important for the survivability of both bacterial and eukaryotic cells but that their antioxidant capacity can be used as a preventative measure against age-related and disease-related damage.

## 4. Methionine Modulating Cellular Activity

Beyond just protecting cells from oxidative stress, the reversible nature of methionine oxidation led to questions regarding whether it could serve as a mechanism of cell signaling and regulation. Early assumptions were that, in most cases, the oxidation of protein-bound methionine was detrimental to protein function [29]. It was not until almost a decade later that the oxidation of methionine was found to be able to activate and deactivate some proteins as a sort of on-off switch to maintain homeostasis [17]. Since then, a plethora of examples of the oxidative state of methionine being involved in the activity state of cells have been identified.

### 4.1. Methionine and Calmodulin

**Figure 5:**
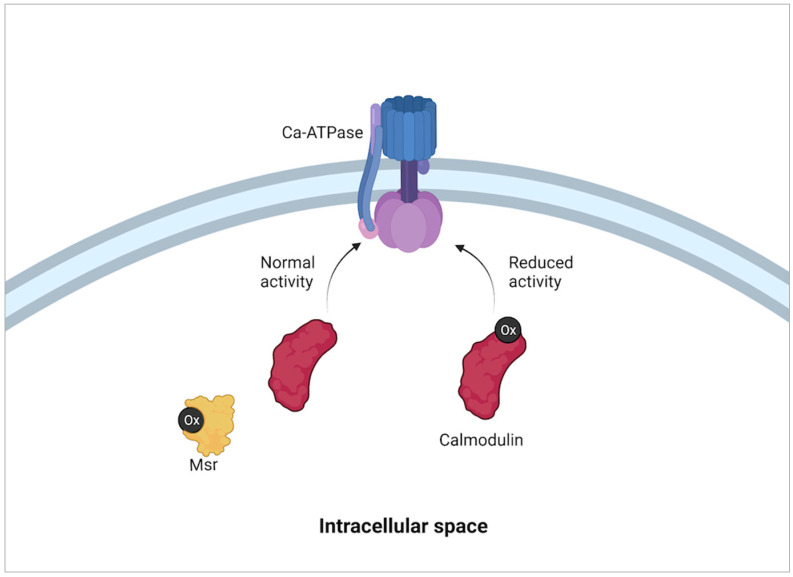
Modulation of calmodulin activity with oxidative state. When reactive oxidative species (Ox in black) concentration is high in a cell, the oxidation of methionine residues within calmodulin modifies its activity. This in turn reduces the activity of Ca-ATPase pumps embedded within the plasma membrane. The reduction of Ca-ATPase pump activity alters intracellular Ca balance which then causes a reduction on cell metabolism. The reduction of methionine residues on calmodulin by methionine sulfoxide reductases (Msr) then returns normal activity to calmodulin and therefore cell Ca-ATPase pumps and cell metabolism [53].

Calcium regulatory proteins such as calmodulin (CaM) and plasma membrane Ca-ATPase (PM-Ca-ATPase) during states of oxidative stress have been observed to have reduced activity associated with the oxidation state of their methionine residues [53] (Figure 5). The reduced activity in the presence of high levels of reactive oxidative species (ROS) is believed to be a survival mechanism as it prevents the production of additional ROS created as by-products of cellular metabolism. Calmodulin has additional multifunctional activity within a cell as a part of important calcium-regulated signal transduction pathways so the discovery of oxidative activity on methionine within CaM led to further inquiries. It was found that the Met77 of CaM was susceptible to oxidation mediated by MsrA which was found to regulate its interaction with one or more of its targets [54]. Further testing using a mutant M77Q which mimicked a perpetually oxidized Met77 revealed that this mutant CaM was less effective in activating CaMKILα [55]. The identification of a direct correlation between CaM oxidation and decreased activity is evidence and confirmation of the modulatory ability of methionine oxidation with CaM which changes cellular function.

### 4.2. Methionine and Actin

Another target for methionine oxidation that has a regulatory effect is the actin Cytoskeleton [56]. The polymerization and depolymerization of actin within cells is a highly regulated process. Of the 16 methionine residues found in actin, 6 have been found to convert to methionine sulfoxide in the presence of oxidizing agents: Met44, Met47, Met176, Met190, Met269, and Met355. Further, Met44 and Met47 exhibit the greatest vulnerability to oxidation [57]. Ruei-Jiun Hung and collaborators used oxidizing proteins known as MICALs [58] to oxidize the Met44 of actin which led to the filament severing and a decrease in polymerization ability [59]. More recently it has been discovered that this oxidation leading to reduced polymerization is a reversible process. The oxidation of Met44 is stereospecific generating the R-MetO form of methionine sulfoxide which can be reduced back to methionine by MsrB methionine sulfoxide reductase [60,61]. The implications of the oxidative and reductive states of F-actin are a capability to modify the polymerization to G-actin thus changing cytoskeletal activity based on the needs of the cell.

### 4.3. Methionine and Ion Channels

The redox potential of the sulfur-containing amino acids also has an effect on the activation states of certain ion channels which control the excitability state of the cell. In certain cases, the oxidation of cysteine and or methionine within Ca^2+^ activated K^+^ channels (KCa) have shown to decrease channel activity with restored upon reduction [62,63]. Interestingly, there have also been examples found of the opposite effect occurring where KCa channels were activated upon oxidation [64]. Though in these cases the redox effect was not confirmed to be caused by either cysteine or methionine specifically, there have been more recent studies where channels that activate and deactivate depending on whether cysteine or methionine is oxidized [65]. In Na^+^ channels, the oxidation of Na^+^ channels in the skeletal muscle and cardiomyocytes of mammals inhibited their inactivation [66]. The inhibition of inactivation in Na^+^ channels of muscle cells can result in changes in the electrical activity of cells, which in the case of cardiomyocytes can lead to the fatal prolonging of action potentials [67,68].

Though these are only a few of the many examples of modification of cellular activity based on methionine’s oxidative state, they exemplify that cells have evolved to use the oxidative capacity of this sulfur-containing amino acid for a number of functions. Since in all of these cases the reduction and therefore resumption of activity following methionine oxidation is dependent on the activity of methionine sulfoxide reductase, it has been postulated that this mechanism of oxidation and reduction could serve as a longer-lasting post-translation modulator of cell activity [69]. Many of the scenarios that have been described are of a negative feedback mechanism that downregulates the activity of cells during times of oxidative stress to prevent further damage. It is evident now that methionine not only serves as an antioxidant but also plays an important part in the balance of cellular activity.

## 5. Cysteine Sharing Methionine Function

### 5.1. Transmethylation and Transsulfuration

**Figure 6:**
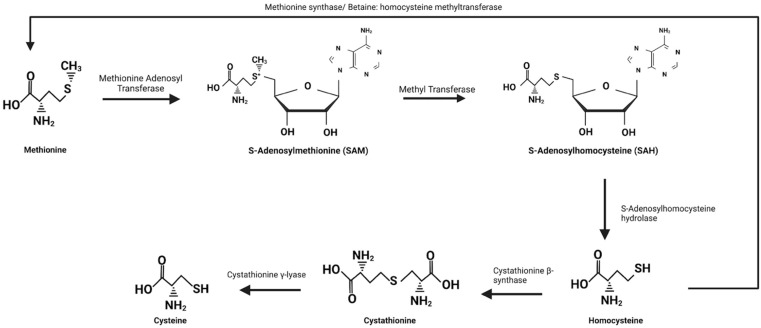
The conversion of methionine to homocysteine and cysteine. Conversion of methionine to S-adenosylmethionine (SAM) begins with methionine adenosyl transferase which is an ATP-dependent reaction requiring the removal of all three phosphate groups from ATP. SAM then donates its methyl group to a methyl acceptor using a methyl transferase to form S-adenosylhomocysteine (SAH). SAH is hydrolyzed into homocysteine and adenosine by S-adenosylhomocysteine hydrolase which marks the end point of the transmethylation reactions. At this point homocysteine may be methylated by either methionine synthase or betaine: homocysteine methyltransferase to be converted back into methionine or undergo transsulfuration to form cysteine. Transsulfuration begins with the conversion of homocysteine to cystathionine by cystathionine β-synthase and then ends with the formation of cysteine from cystathionine by cystathionine γ-lyase.

The expansive role of methionine within proteins has been the subject of recent work to exemplify that it is indeed important beyond the initiation of protein synthesis. However, whether these functions are essential or merely useful adaptations is of interest. Methionine and cysteine have a close association within cells which is well demonstrated by the transmethylation and transsulfuration pathways. The relationship between methionine and cysteine is a balance, with methionine being used for the synthesis of cysteine. An early study by Vincent du Vigneaud and colleagues demonstrated the metabolic conversion of methionine to cysteine using radiolabeled sulfur and found that 80% of sulfur found in cysteine had been derived from the labeled methionine [70]. What was later discovered by Cantoni was the intermediate S-adenosylmethionine (SAM) which aided in the understanding of the pathway of methionine metabolism [71]. The first series of reactions, known as transmethylation, begins with the conversion of methionine to SAM which is completed by the enzyme methionine adenosyl transferase. The newly formed SAM then utilizes a methyl transferase to donate its methyl group to an acceptor which results in the formation of an additional intermediate: S-adenosylhomocysteine (SAH). SAH is then hydrolyzed by the enzyme S-adenosylhomocysteine hydrolase which produces homocysteine marking the end of the transmethylation pathway. Homocysteine lies at a key junction in the metabolic pathway between methionine and cysteine. Depending on cysteine and methionine levels in the cell, homocysteine may be methylated back into methionine by methionine synthase, or it may undergo transsulfuration to produce cysteine (Figure 6). In studies where excess cysteine was supplemented, parenteral methionine supplemental requirements dropped to 70% of the enteral requirements which was coupled with an increase in the re-methylation pathway of homocysteine [72,73]. This highlights the interconnection and internal balance that is maintained between these two amino acids and that cysteine production by methionine is not an essential function of methionine.

An important characteristic of this pathway is that, while homocysteine can be converted to either methionine or cysteine, once homocysteine undergoes transsulfuration it cannot be converted back to methionine. This principle relates to the study by Madelyn Womack and colleagues because cysteine cannot be converted to methionine so, with only cysteine supplementation, protein synthesis was unable to be initiated at optimal rates, and growth was significantly reduced [19]. Again, referring to the work of Madelyn Womack and William Rose, the observation of normal growth in animals supplemented with suboptimal methionine and excess cysteine provide the basis of the theory that all of the previously discussed roles of methionine are non-essential beyond the initiation of protein synthesis [20].

### 5.2. Cysteine and Methionine in Protein and Cell Structure

When looking at the structural contributions that both of these amino acids provide to proteins, there are many similarities. Similar to methionine, the sulfur atom of cysteine is subject to oxidation and, once oxidized, cysteine has the ability to form disulfide linkages within a protein. These disulfide linkages help to stabilize the tertiary and quaternary structures of proteins and exogenous proteins with more disulfide linkages are able to maintain biological function in more extreme environments [74]. Though this ability does showcase an example of cysteine’s protein-stabilizing properties, it is only relevant for extracellular proteins as the formation of disulfide bonds is restricted within intracellular proteins [75]. Relevant to intracellular proteins is both the hydrophobic property of cysteine as well as its potential to form S-aromatic bonds similar to methionine. The hydrophobic nature of cysteine was first described by Nozomi Nagano and coworkers who found that free cysteine residues were located in the hydrophobic core of 3D-modeled proteins with the likes of methionine [36]. Cysteine is unique as well in that its side chain provides both hydrophobic and polar properties. The polar nature of cysteine would seem to directly contrast its contribution to the hydrophobic core of proteins, but recent evidence has shown that the hydrophobic properties of cysteine are energetically favorable to its polar nature [18].

Additional evidence that experimentally cysteine may serve as a replacement for methionine emerges when considering the S-aromatic motifs. While it was a valuable protein-stabilizing feature of methionine inclusion, it is not unique to methionine. S-aromatic interactions are simply non-covalent complexes formed between aromatic residues and sulfur-containing groups. Cysteine has been well documented to be able to form these motifs within proteins [25,76,77]. Given the hydrophobic nature of cysteine as well as the ability to form S-aromatic motifs, it certainly suggests that on the basis of the protein stabilizing action of methionine, cysteine can serve this same role in methionine’s absence.

### 5.3. Cysteine’s Antioxidant Function

The endogenous antioxidant feature of methionine, much like the S-aromatic motifs, is centered around methionine’s sulfuric side chain. As such, cysteine shares many of these antioxidant abilities and some that go even beyond sulfur oxidation. Cysteine differs from methionine in that, the sulfur of cysteine is in a thiol form compared to methionine’s thioether form. The functional significance of the thiol form on cysteine is that it is ionizable and contains a negative charge that is generated after deprotonation thus boosting its reactivity. Additionally, this thiol group is subject to alkylation and is regularly targeted for oxidation by reactive oxygen and nitrogen species [78]. The incorporation of cysteine into the active sites of proteins such as thioredoxin (Trx), peroxiredoxin (Prx), and glutaredoxin (Grx) is what gives these proteins the ability to detoxify ROS and reduce oxidized protein thiol residues. Of these three proteins, peroxiredoxin is the most significant contributor in antioxidant defense due to its abundance and high rate constant [79]. The cysteine within peroxiredoxin is well conserved and serves as the site of oxidation by peroxides. When oxidized, this cysteine forms cysteine sulfenic acid which then reacts with an additional cysteine residue within peroxiredoxin to form a disulfide bridge that is then reduced to complete the cycle [80]. The rate at which cysteine in peroxiredoxin is oxidized is about 1 × 106 to 108 M^−1^s^−1^ which is 5 to 7 magnitudes higher than the rate of other small thiols [80]. Trx is unique in that its importance is not due to its own antioxidant capabilities, but rather because of its role in other antioxidant systems. Trx plays a role in both the Prx antioxidant system as well as the previously mentioned methionine sulfoxide reductase function by reducing disulfide bridge formation within these proteins, thus recycling their antioxidant function [81]. The third of the cysteine dependent antioxidant proteins mentioned is glutaredoxin which uses glutathione (GSH) as a cofactor. Glutathione is a tripeptide that consists of cysteine, glutamic acid, and glycine and is present in most mammalian tissue. It serves as a free radical scavenger and detoxifying agent that protects proteins against oxidation via ROS and the reduction of glutathione increases oxidative stress within an organism [82]. Glutaredoxin differs from thioredoxins in that there is no specific oxidoreductase responsible for reducing glutaredoxin, rather, glutaredoxin will be reduced through the oxidation of the GSH [83]. Grx does however function in a similar capacity to Trx by the reduction of disulfide bridges formed within antioxidants such as ascorbic acid. These three examples exemplify the use of cysteine in a number of crucial proteins responsible for protecting cells from oxidative damage, yet only represent a part of the antioxidant function of cysteine.

Much like methionine residues on the surface of proteins serving as endogenous antioxidants, there is evidence of similar abilities by cysteine residues. The concept of oxidation of thiol residues on proteins as an endogenous antioxidant was first discussed by James Thomas and colleagues but had little experimental evidence to back the claim [84]. The common understanding had long been that glutathione was the predominant intracellular thiol, though experiments measuring thiol abundance pointed toward protein thiols as being the prevailing intracellular thiol [85]. In a groundbreaking study conducted by Raquel Requejo and lab members, it was discovered that the majority of free thiols in rat liver and heart cells were from surface-exposed cysteines and not glutathione-incorporated cysteines [86]. With this, it was confirmed that the majority of cysteine thiol residues are found on the surfaces of proteins rather than in antioxidant proteins such as GSH. When comparing cysteine and methionine on the basis of antioxidant capabilities, there is clearly a parallel in their function on the surface of proteins. However, cysteine’s antioxidant contribution extends beyond this as it is the functional component of a number of antioxidant and reducing proteins both responsible for protecting against ROS directly and recycling the antioxidant capabilities of other proteins.

### 5.4. Cysteine Modulating Cell and Protein Activity

**Figure 7:**
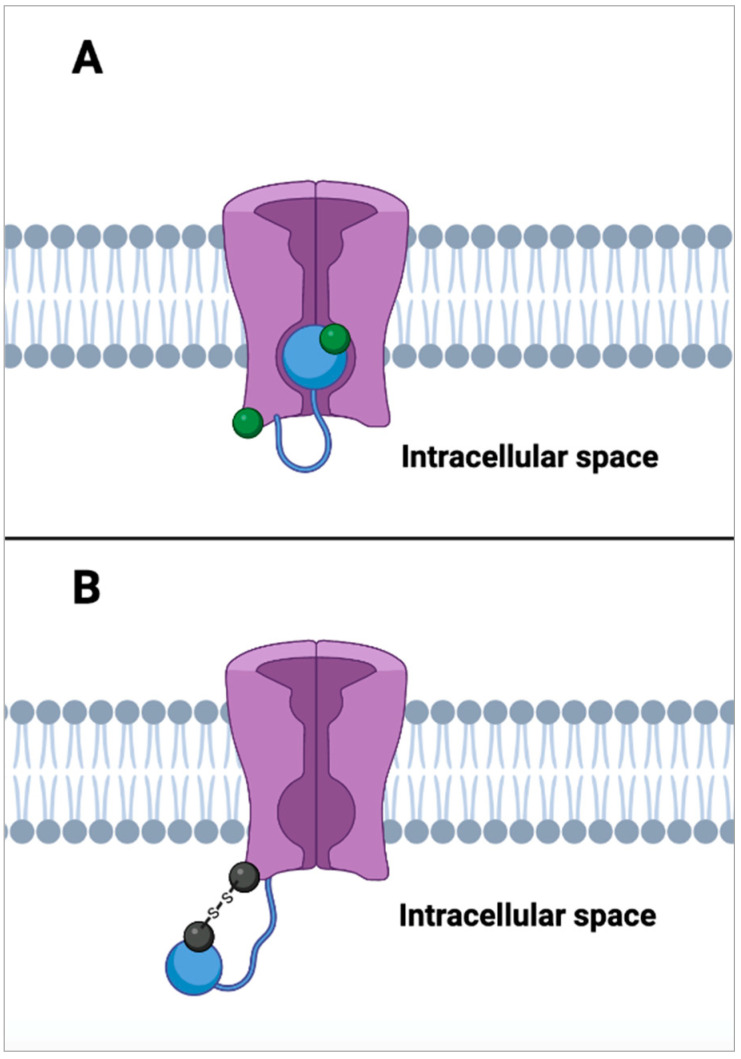
Inhibition of voltage-gated potassium channel inactivation. (**A**) shows a voltage gated K^+^ channel (in purple) in a state of inactivation. This state occurs when an auto-inhibitory peptide (blue ball) that is tethered to the N-terminus of the K^+^ channel binds to the open channel pore by competing with intracellularly applied channel blockers [90]. When cysteine residues located on both the blocking peptide and the channel itself are in their reduced state (green ball), inactivation activity is rapid. (**B**) shows the inhibition of inactivation by the auto-inhibitory peptide. When the cysteine residues on the inhibitory peptide and channel are oxidized (black ball), they form disulfide bridges. These disulfide bridges effectively tether the auto-inhibitory channel blocking peptide to the N-terminus of the K^+^ channel which prevents it from blocking K^+^ ion flow leaving the channel in a state of activity [87].

When looking at the modulatory ability of cysteine oxidation and reduction, there are a number of important examples that have been noted. Similar to methionine oxidation affecting ion channel activity, the oxidation of cysteine has been observed to be an inhibitor of fast inactivation by preventing the closure of the pore by the gate mechanism of K^+^ channels [87]. This is due to the formation of disulfide bridges between cysteine residues on the channel and cysteine residues within the inactivation gate maintain an open conformation (Figure 7). Adding on to these findings, work by Stefan Heinemann and colleagues found evidence for cysteine oxidation and reduction controlling inactivation within the ß subunits as well [88]. This very closely mirrors the disruption of inactivation for K^+^ channels detected by the oxidation of methionine residues [89], which implies that these mechanisms may work in concert but also be able to function independently with little change in the modulatory effects on these channels.

Another significant example of cysteine oxidation regulating enzymatic activity was identified within the TCA cycle of the mitochondria. A study by Danilo Daloso and contributors explored the factors contributing to the flux of the plant mitochondrial tricarboxylic acid (TCA) cycle [91]. The target of this study was the redox protein thioredoxin which is present in all organisms and known to reduce oxidized cysteines and cleave disulfide bonds. Using double mutant, double knockout plants lacking two genes, ntra and ntrb, which encode for NADP-thioredoxin reductase a and b as well as inserting a mutant thioredoxin o1 gene, researchers found a corroborating effect for thioredoxin in regulating the TCA cycle. The presence of thioredoxin was associated with the deactivation of succinate-dehydrogenase and fumarase as well as the activation of ATP-citrate lyase. These findings were interpreted by researchers as evidence that thioredoxin-mediated thiol switching has a significant impact on regulating the TCA cycle. Though this experiment was conducted using a plant model, these findings may be considered relevant in non-plant mitochondria due to the conservation of specific cysteine residues in citrate synthase, NAD-dependent isocitrate dehydrogenase, fumarase, malate dehydrogenase, and aconitase [92]. What this study shows is yet another example of cysteine’s redox activity being used directly as a control mechanism of enzymatic activity with overarching effects on mitochondrial and cellular metabolism.

**Figure 8:**
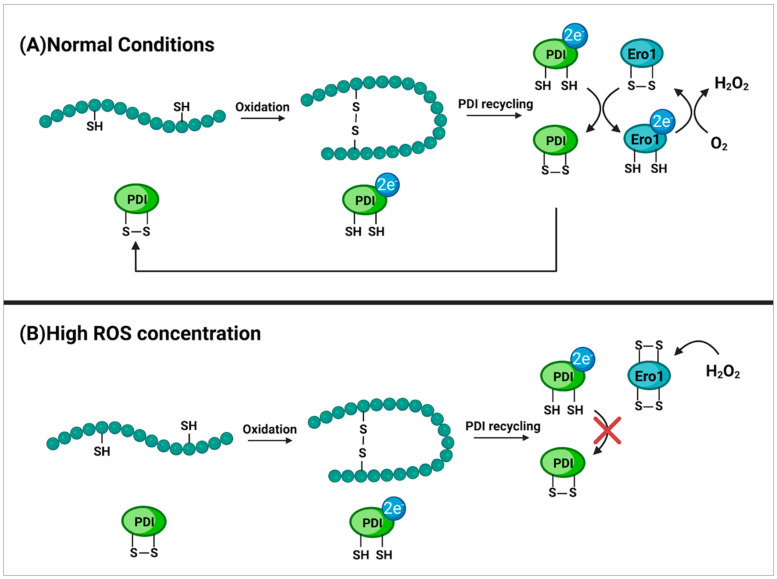
Cysteine oxidation of Ero1 as a protection mechanism. (**A**) displays the formation of disulfide bridges within proteins bound for the extracellular environment. This takes place in the endoplasmic reticulum of the cell and is mediated by the protein disulfide isomerase (PDI). Upon disulfide bridge formation, PDI is reduced and must transfer its electrons to the disulfide oxidase enzyme endoplasmic reticulum oxireductin 1 (Ero1) in order to return to its active state. The now reduced Ero1 then transfers electrons to a terminal acceptor oxygen which returns Ero1 to its active state and forms H_2_O_2_ as a byproduct [93]. (**B**) shows the formation of intramolecular disulfide bridges between cysteines within Ero1 due to high concentrations of H_2_O_2_. This inhibits regular activity of Ero1 which then in turn prevents the recycling of PDI and therefore the formation of disulfide bridges within proteins. This serves as a protection mechanism to prevent the over-production of reactive oxidative species (ROS) during times of oxidative stress in order to protect the cell from damage [94,95,96].

As a final example of the diversity of known locations in which cysteine’s redox potential is utilized to control enzymatic activity, its function in the endoplasmic reticulum is crucial for protection under high oxidative stress. Exogenous proteins released by the ER form disulfide bonds before they are released to improve protein stability in the exogenous environment. The formation of these bonds is within the ER is catalyzed by protein disulfide isomerase (PDI) and endoplasmic reticulum oxireductin 1 (Ero1). PDI oxidatively folds extracellular bound proteins and oxidase transfers electrons to Ero1 which then transfers those electrons to a terminal acceptor oxygen. The transferring of electrons to oxygen by Ero1 produces a single molecule of H_2_O_2_ per de-novo disulfide bond formed [93]. In order to prevent the overproduction of oxidative H_2_O_2_, cysteine residues within the Ero1 will form intramolecular disulfide bonds during times of high H_2_O_2_ production which constrict Ero1 activity and therefore reduce the oxidative stress within the cell [94,95,96] (Figure 8). These findings all show that it is the redox capacity of cysteine that is controlling the activity in these different structures, much like what was observed in methionine modulated activity. To date, the replacement of methionine residues with cysteine residues in a selected protein has not been attempted. However, due to methionine and cysteine’s shared hydrophobic nature, ability to form S-aromatic motifs, endogenous antioxidant potential, and modulatory effects via their reversible oxidative states, it may be possible that little change to the selected protein’s function would occur.

## 6. Alternative Mechanisms for Protein Synthesis Initiation

### 6.1. Initiating Protein Sythesis with Alternative Amino Acids

The potential for cysteine to serve in place of methionine in the previously mentioned functions in a cysteine/methionine substituted protein seems possible. If this is indeed the case, the true essential nature of methionine seems to be within the context of protein synthesis initiation. However, in recent years, evidence has emerged challenging even this notion and suggesting alternative initiators of protein synthesis. Methionine has been known as the initiator of protein synthesis for a number of decades, and during this time, researchers have defined the features of methionine and formyl methionine tRNA that give it this unique ability as is well described in work by Christine Mayer and collaborators [97]. Modern studies have used this information in an attempt to identify amino acids with similar properties and manipulate these structures that could serve as a replacement for methionine tRNA in vivo. In *E. coli*, substitutions of the methionine initiating anti-codon CAU with alternative anti-codons have shown an ability to initiate protein synthesis using valine, phenylalanine, isoleucine, tryptophan, and glutamine [98,99,100,101]. In a recent review [102] and subsequent experiments [103,104], the initiation of protein synthesis using non-canonical amino acids with mis-acetylated formyl methionine tRNAs both in vivo and in vitro is shown to be possible. These findings are very new; however, they display incredible plasticity and adaptability of translation machinery.

As methionine lies at a critical junction of the central dogma of eukaryotic translation, it was not until the functional adaptability in bacterial translation was identified that this mechanism in eukaryotes was tested. Among the first of these studies in eukaryotes was done by Harold Drabkin and Uttam Rajbhandary where they identified mutant initiator tRNAs, AGG and GUC which were able to initiate protein synthesis using methionine and valine respectively [105]. Though mutagenesis of these alternative initiator tRNAs was completed by experimenters, the significance of these results is that there were no downstream detrimental effects on the structure or function of proteins produced or cell viability in the case of valine substitution. In a similar manner that *E. coli* translational machinery showed incredible adaptability during initiation, it is evident that eukaryotic cells share this trait.

### 6.2. Ribosome Reinitiation

Another potential mechanism of interest is the reinitiation of ribosomes following the termination of elongation. The normal sequence of events upon arrival of an elongating ribosome at a termination codon is the recruitment of release factors eRF1 and eRF3 which complex with GTP and bind to the A-site of the ribosome [106]. These factors trigger the release of the completed protein product which is then followed by the release of the 60S ribosomal subunit, ejection of the deacylated tRNA on the small 40S subunit, and finally, the ejection of the 40S subunit itself. However, what has also been found is that these same ribosomes may undergo reinitiation due to the incomplete recycling of the ribosomal complex on an upstream open reading frame (uORF) [106]. The reinitiation process is generally dependent on the presence of an uORF, which is defined as an initiation codon and a termination codon separated by at least one additional sense codon. The presence of uORFs within transcripts is quite common with a ribosome profiling assay of human transcripts detecting the presence of uORFs in more than 50% of the transcriptome [107] and nearly 61% of zebrafish transcriptome showed active translation of detected uORFs [108]. With a large presence of these uORFs present in the eukaryotic transcripts, it would appear that reinitiation of ribosomes is a fairly common and important mechanism. It is known that uORFs have a significant impact on the transcription of thousands of human genes, and therefore play a large part in the regulation of transcription. It is known that under unstressed conditions, uORFs correlate with significantly reduced protein expression of downstream ORFs [109]. What is even more interesting is the fact that, under environmental stress, uORFs will cause an increase in the expression of genes related to environmental stress protection [110]. Further insight into the mechanism by which reinitiation of the ribosome occurs found that reinitiation-promoting elements will interact with eIF3 on the early elongating ribosome. These elements will cause eIF3 to remain intact on the ribosome and stabilize the post-termination complex in order to promote reinitiation of the downstream codon [111]. This further characterization of the components relevant to ribosome reinitiation and the discovery of uORFs association with environmental stress genes shows yet again another non-traditional route of initiation of protein translation. Relating these findings to the topic at hand, in the presence of a mutant initiator tRNA in a methionine deficient environment, uORFs may promote the translation of genes involved in cell survival under stressed conditions.

### 6.3. Internal Ribosome Entry Sites

**Figure 9:**
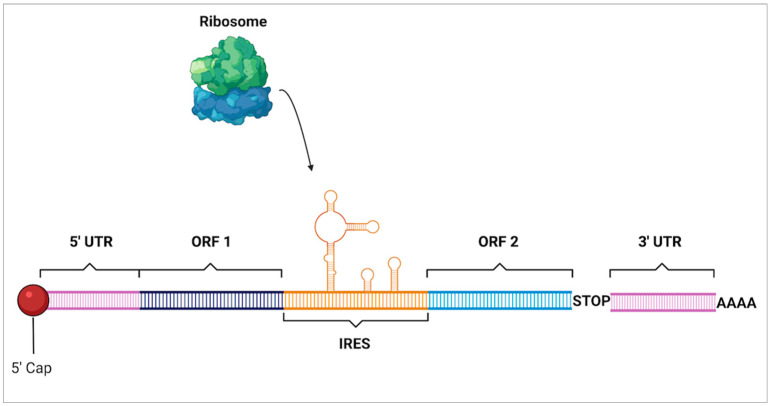
Internal ribosomal entry site. Internal ribosomal entry sites (IRES) are complex secondary structures that form within mRNA that allow mammalian ribosomes to bind and begin translation of open reading frames (ORF) in a cap-independent manner which bypasses traditional initiation at the 5’ cap near the 5’ untranslated region (UTR).

An additional mRNA motif that is of interest is the internal ribosomal entry sites (IRES) (Figure 9). These are RNA structures located on the 5′ end of untranslated regions within polycistronic mRNAs. They have a diversity of forms which can range from 9 to 1000 nucleotides in length as well as being unstructured or forming secondary and tertiary structures [112]. IRES are able to bypass the typical scanning phase of the ribosomal initiation complex so that initiation can occur independently of the 5′ cap of mRNA. These complexes also remodel the small ribosomal subunit which then allows it to include the internal template into the RNA-binding channel [113]. This mechanism is commonly utilized by viruses which will suppress the normal translational activity of the host cell and then bind to the IRES to monopolize translation to only viral particles. While viruses are where IRES were discovered, it is now understood that mammalian cells take advantage of them as well. Under stressed conditions that downregulate cap-dependent translation such as hypoxia and nutrient deficiencies, cancer cells have been seen to use IRES to continue translation [114]. Later, the identification of thousands of viral and human sequences that initiate translation using IRES which is essential both in stressed conditions as well as during normal development [115]. Though most of this research is very new, it uncovers that there are an increasing number of motifs that may be utilized to continue translation in both nutrient-deficient environments as well as in non-stressed conditions.

## 7. Conclusions

As the properties of methionine continue to be explored it is clear that the uses for methionine go beyond simply initiating protein synthesis. The ability of the sulfur atom within methionine to form S-aromatic motifs within proteins has been shown to be a significant stabilizing factor helping to prevent protein denaturation. Evidence of methionine as a compelling antioxidant has not only showcased that the replacement of methionine with norleucine increased oxidative stress within cells but also that cells have adapted to take advantage of methionine’s antioxidant properties by adding methionine residues around vulnerable receptors. Methionine has also been found to be a potent regulator of enzymatic activity once again highlighting that methionine serves a number of vital protective roles within cells. However, though cells utilize methionine for a growing variety of functions, they also maintain the ability to adapt to environments where methionine may not be present. Many of the functions of methionine center around its inclusion of a sulfur atom: a feature that is shared with cysteine. Cysteine is also able to form stabilizing S-aromatic motifs in intracellular proteins as well as disulfide bonds in extracellular proteins, and with the hydrophobic nature of cysteine having recently been identified, it may likely be able to serve a similar stabilizing function as methionine within the protein core. The antioxidant features of cysteine seem to be even further reaching than that of methionine as cysteine has the same intrinsic antioxidant ability granted to it by a sulfur atom but with the added factor that it is essential for the synthesis of the intracellular antioxidant glutathione. Though the examples of methionine and cysteine modulating cell activity were not identical, the identification of their redox states correlating to inhibition or activation of proteins suggests they function similarly. Due to recent exploration into the mechanisms initiating protein synthesis, it now seems possible that in the absence of methionine that cells could use an alternative initiating amino acid or even circumvent the initiation process by using unique structural motifs within mRNAs. Though maximal cell and protein function is under conditions where all amino acid requirements are met, both the bacterial and eukaryotic cells used in experimentation have shown impressive plasticity under environmental stress. The research suggested based on the points made in this review are two-fold: (I) to use mutant methionine tRNA which charges with cysteine and observe the impact on function of the selected and (II) based on the findings of that study, use one of the identified alternatives to traditional translation initiation to attempt the complete synthesis of a functional protein completely free of methionine. The outcome of these studies could be used to identify the essentiality of these newly identified methionine functions as well as further characterize alternative translation initiators in eukaryotic cells. The compilation of findings within this paper highlights what is an actively evolving understanding of not only methionine’s role within proteins and cells, but also the complex and adaptive nature of the translational machinery of the cell.

## Data Availability

Not applicable.
